# Everybody's Page

**Published:** 1891-05-23

**Authors:** 


### Everybody's Page.

The natural inference to be drawn from the manifest predilection of the present epidemic of influenza for people of rank is that this particular microbe is a bit of a snob.

People who are unfortunate enough to suffer from the disfigurement of red hands may be tempted to use the following ointment which is said to be a good remedy: Lanolin, 2 drams; liquid paraffin, 5½ drams; rosemary oil, 2 drops. The hands should be first washed with a lathering soap and carefully dried.

According to the *American Druggist*, the weather this spring in Canada has been unusually unpleasant. The bottom has completely dropped out of business. Last year everybody bought grippe medicine, even those who were well supplied themselves with prophylactics. "But this year you can't talk a man into taking even an almanack with a place for memoranda and cuts of grateful patrons on each page."

Professor Angell, of Michigan University, gives the following simple test for drinking water which may be of use to the householder whether in town or country: "Dissolve half a teaspoonful of the purest white sugar in a pint bottle quite full of the water to be tested and stop the bottle carefully; then expose it to daylight and to a temperature up to 70° F." If examined after a day or two, when held against something black, any organic matter present in the water, will appear in the form of floating specks.

In one respect at least we are wiser than our cousins on the other side of the water. Drunkenness is not a punishable offence in this country, where unless a drunken man molests his fellow citizens he is only arrested for his own protection whilst incapable and given time to reflect upon the ridiculous and often painful exhibition he has made of himself. In America the punishment of inebriates as criminals, which is considered by some to have a deterring effect, too often results in making the drunkard really a criminal and a needless cost to the community.

The American Association for the Cure of Inebriety has taken alarm, as it well may do, at the increasing consumption of morphine, chloral, and cocaine. As the law stands at present, there is nothing to prevent people from indulging to the top of their bent in these narcotics and laying up store for a miserable ending. The only way out of the difficulty which commends itself to the Association is to promote legislation making it illegal for chemists to sell these drugs except in medical prescriptions which cannot be repeated without the written order of a doctor.

There is a most refreshing simplicity about the method alleged to be employed by a modern alchemist in his mystic dealings. The credulity of human nature offers a wide field for the fertile and ingenious brains of those gentlemen who live by the successful accomplishment of the confidence trick. Yet it is hard to imagine anyone possessing such faith in the discovery of the philosopher's stone as to intrust his gold to a gentleman who declares that he will convert it into an untold sum, and who proceeds to make such a diabolical smell with his crucibles that no one can stay in the room with him. Then the alchemist, it is alleged, seizes his opportunity and departs—with the gold! Perhaps it would be nearer the mark to say that avarice rather than credulity is the motive with such people, who are worthy of little sympathy in their loss.

The old woman who praised with such enthusiasm the sermons of a Scotch divine who had acquired a great name for depth and sublimity, would no doubt for similar reasons be an ardent admirer of certain poets. When questioned as to whether she understood the minister, "Understand him!" she ejaculated, holding up her hands in astonishment at such a question; "Me understand him! Wad I hae the presumption?"

It behoves London ratepayers to watch assiduously and jealously the progress of the Water Supply Bill, which is now before a Select Committee of the House of Commons. The amount of money to be paid will be very large, and it depends much upon the provisions relating to purchase whether the future lot of that unfortunate beast of burden—the ratepayer—is to be a happy oneor not.

It appears from evidence given before the Committee of the House of Lords on Child Insurance that the greatest amount of business is done in Dublin among the artizans and petty shop-keeping classes, amongst whom there is very little abuse of the practice; the greatest offenders are to be found among general servants and labouring classes, which is only one more fact pointing to the close association between ignorance and crime.

Now that the great Koch bubble has burst, and tuberculosis still holds its ground, people are beginning to question whether it would not be wiser and more logical to try not so much to combat an accomplished evil as to find means of overcoming the predisposition which exists in more or less pronounced degree in some individuals to acquire the malady, and, like all true sanitarians, pin their faith on prevention rather than cure.

The frequenters of some of the cheap restaurants of our large towns would probably suffer from a painful falling-off in the appetite were they first to pay a visit to the kitchens from which their savoury morsels come. In the interests of their hunger they doubtless show sound discretion in taking the goods the gods of the gridiron send them without questioning or demur. On the other hand, in the interest of sanitation and public welfare, it is quite as important that the cleanliness of our restaurant kitchens should be beyond suspicion as that of our baking establishments. The local authorities are empowed by the Sanitary Acts to deal with any work-place which is not kept in a cleanly state, or not properly ventilated, &c., so as to be injurious or dangerous to health. It would be interesting to know what attention the local authorities have shown to these establishments.

How to start in life advantageously the children who have been brought up in reformatory and industrial schools has always been a difficult problem for the managers of these institutions to solve, and the difficulties they have to meet are only too frequently aggravated by the position taken up by the parents who veto the opportunities that arise. How essential it is to give children, if possible, a good start is shown by the fact that of the boys returning home from the Middlesex Industrial School 16 per cent. relapse into crime, whereas of those provided by the managers with situations only 6 per cent. do so. Mr. Howard Vincent is anxious to get rid of the parents' rights, and to make it legal for the managers of the schools to place in any trade or calling boys who have conducted themselves well, and are willing to enter such trade. In the event of a child being disposed of by emigration he would protect the parents by making the consent of the Home Secretary a necessary preliminary.

